# Prevention and Control of Seasonal Influenza with Vaccines: Recommendations of the Advisory Committee on Immunization Practices — United States, 2025–26 Influenza Season

**DOI:** 10.15585/mmwr.mm7432a2

**Published:** 2025-08-28

**Authors:** Lisa A. Grohskopf, Lenee H. Blanton, Jill M. Ferdinands, Carrie Reed, Vivien G. Dugan, Demetre C. Daskalakis

**Affiliations:** ^1^Influenza Division, National Center for Immunization and Respiratory Diseases, CDC; ^2^National Center for Immunization and Respiratory Diseases, CDC.

**Summary**

**What is already known about this topic?**

Influenza vaccination protects against influenza and its potential complications. The Advisory Committee on Immunization Practices makes influenza vaccination recommendations for each influenza season.

**What is added by this report?**

Information for the 2025–26 influenza season includes the updated vaccine composition, approval of FluMist (nasal spray live attenuated influenza vaccine) for self-administration or caregiver administration, expansion of the approved age threshold for Flublok (recombinant influenza vaccine) from ≥18 years to ≥9 years, and a recommendation that only single-dose seasonal influenza vaccines not containing thimerosal as a preservative be used.

**What are the implications for public health practice?**

Routine annual influenza vaccination is recommended for all persons aged ≥6 months without a contraindication to vaccination to protect against influenza and its complications.

## Abstract

This report updates the 2024–25 recommendations of the Advisory Committee on Immunization Practices (ACIP) concerning the use of seasonal influenza vaccines in the United States. Routine annual influenza vaccination is recommended for all persons aged ≥6 months who do not have a contraindication to vaccination. Multiple formulations of the trivalent inactivated influenza vaccines (IIV3s), trivalent recombinant influenza vaccine (RIV3), and trivalent live attenuated influenza vaccine (LAIV3) are expected to be available for the 2025–26 influenza season. Updates for the 2025–26 season include 1) antigenic composition of 2025–26 U.S. seasonal influenza vaccines, 2) Food and Drug Administration (FDA) approval of FluMist (LAIV3) for self-administration or caregiver administration, 3) FDA approval of a change in age indication for Flublok (RIV3) from ≥18 years to ≥9 years, and 4) a new ACIP recommendation that children aged ≤18 years, pregnant women, and all adults receive seasonal influenza vaccines only in single-dose formulations that are free of thimerosal as a preservative. A comprehensive summary of recommendations, including those discussed in this report, as well as previous recommendations concerning topics not addressed in this report and that remain unchanged for the 2025–26 season, is available at Influenza | ACIP Recommendations for Vaccination. Additional background information also is available at Prevention and Control of Seasonal Influenza with Vaccines.

## Introduction

The Advisory Committee on Immunization Practices (ACIP) provides annual recommendations for the use of influenza vaccines for the prevention and control of seasonal influenza in the United States. This report summarizes updates to the 2024–25 recommendations for use of seasonal influenza vaccines in the United States for the 2025–26 influenza season. As in previous seasons, routine annual influenza vaccination is recommended for all persons aged ≥6 months who do not have a contraindication to vaccination. Various formulations of influenza vaccines are available. Contraindications to and precautions for the use of influenza vaccines are summarized. Updated recommendations discussed in this report reflect discussions held during public ACIP meetings on April 15 and June 26, 2025.

This report describes new and updated recommendations for the 2025–26 season, as well as several topics for which recommendations remain unchanged from the 2024–25 season, including populations for whom influenza vaccination is recommended, timing of vaccination, selection of vaccines, and contraindications and precautions. Previous recommendations concerning topics and specific populations not discussed in this report remain unchanged from the 2024–25 season. Additional information on topics not addressed in this report are available the 2024–25 ACIP seasonal influenza vaccination recommendations (*1*). Recommendations and updates included in this report supersede previous recommendations. In addition, a comprehensive summary of the recommendations for the 2025–26 influenza season, including topics covered and not covered in this report, is available at Influenza | ACIP Recommendations for Vaccination.

## Methods

### Influenza Work Group Activities

The ACIP Influenza Work Group meets by teleconference regularly throughout the year to review topics before they are discussed at ACIP meetings. Systematic review and evidence assessment are not performed for changes in the viral antigen composition of seasonal influenza vaccines recommended by the Food and Drug Administration (FDA) and changes that reflect use that is consistent with FDA-approved indications and prescribing information. Systematic review and evaluation of evidence using the Grading of Recommendations Assessment, Development, and Evaluation (GRADE) approach (*2*) are typically performed for new recommendations or substantial changes in current recommendations. Evidence is reviewed by the ACIP Influenza Work Group, and work group considerations are included within the ACIP Evidence to Recommendations (EtR) framework to guide the development of recommendations proposed for a vote by ACIP (*2*,*3*). Because the reaffirmed recommendations for seasonal influenza vaccination had no new recommendations or substantial changes, this recommendation did not require performance of GRADE or EtR. However, GRADE and the EtR framework were used in the development of the previous recommendations mentioned in this report for influenza vaccination of adults aged ≥65 years (*4*,*5*) (June 2022), persons with a history of egg allergy (*6*,*7*) (June 2023), and solid organ transplant recipients aged 18 through 64 years (*8*,*9*) (June 2024). GRADE and EtR were not used to develop the recommendation to avoid seasonal influenza vaccines containing thimerosal as a preservative, and this topic, recommendation, and recommendation language were not discussed by the Influenza Work Group.

### Updates to the Influenza Vaccination Recommendations

Four updates to the 2024–25 recommendations are presented in this report. These include three FDA-approved labeling changes and a new recommendation approved through discussion at the June 2025 ACIP meeting.

1. In March 2025, FDA issued recommendations for the antigenic composition of 2025–26 U.S.-approved influenza vaccines (*10*).
2. In September 2024, FDA approved FluMist (LAIV3) for self-administration (for recipients aged 18 through 49 years) or administration by a caregiver aged ≥18 years (for children and adolescents aged 2 through 17 years). FluMist for self-administration or caregiver administration is anticipated to become available during the 2025–26 season (*11*).
3. In March 2025, FDA expanded approval of Flublok (RIV3), previously approved for persons aged ≥18 years, to children and adolescents aged 9 through 17 years. Flublok is now approved for persons aged ≥9 years (*12*).
4. On June 26, 2025, ACIP made a new recommendation that children aged ≤18 years, pregnant women, and all adults receive seasonal influenza vaccines only in single-dose formulations that are free of thimerosal as a preservative.

## Vaccine Composition for the 2025–26 Influenza Season

Based on review of U.S. and global influenza surveillance data (*10*), all influenza vaccines available in the United States during the 2025–26 season will be trivalent vaccines containing hemagglutinin derived from 1) an influenza A/Victoria/4897/2022 (H1N1)pdm09-like virus (for egg-based vaccines) or an influenza A/Wisconsin/67/2022 (H1N1)pdm09-like virus (for cell culture–based and recombinant vaccines); 2) an influenza A/Croatia/10136RV/2023 (H3N2)-like virus (for egg-based vaccines) or an influenza A/District of Columbia/27/2023 (H3N2)-like virus (for cell culture–based and recombinant vaccines); and 3) an influenza B/Austria/1359417/2021 (Victoria lineage)-like virus. This composition reflects an update in the influenza A(H3N2) component compared with that contained in the vaccines during the 2024–25 influenza season (*10*).

## Recommendations for Influenza Vaccination for the 2025–26 Season

Routine annual influenza vaccination of all persons aged ≥6 months who do not have a contraindication to vaccination continues to be recommended. Information on timing, selection, administration, and contraindications and precautions follows.

### Timing of Vaccination

For most persons who require only 1 dose of influenza vaccine for the season, vaccination should ideally be offered during September or October. However, vaccination should continue after October and throughout the influenza season as long as influenza viruses are circulating and unexpired vaccine is available.

Vaccination during July and August is not recommended for most groups because of potential waning of vaccine-induced immunity during the influenza season (*13*–*33*), particularly among older adults (*13*,*14*,*16*,*23*,*26*,*32*). However, vaccination during July or August may be considered for any recipient if there is concern that later vaccination might not be possible. Considerations for timing of vaccination follow.

- **Most adults (particularly those aged ≥65 years) and pregnant women in the first or second trimester.** Vaccination during July and August should be avoided unless there is concern that vaccination later in the season might not be possible.
- **Children who require 2 influenza vaccine doses.** Children aged 6 months through 8 years who did not receive ≥2 trivalent or quadrivalent influenza vaccine doses before July 1, 2025, or whose influenza vaccination history is unknown, require 2 doses of influenza vaccine for the season (Supplementary Figure). These children should receive their first dose as early as possible (including during July and August, if vaccine is available) to permit receipt of the second dose (which must be administered ≥4 weeks later), ideally by the end of October.
- **Children who require only 1 influenza vaccine dose.** Vaccination during July and August can be considered for children of any age who need only 1 dose of influenza vaccine for the season. Although waning of immunity after vaccination during the season has been observed among all age groups (*13*–*33*), fewer studies report this specifically among children (*13*,*22*,*24*,*25*,*29*,*31*,*32*). Moreover, children in this group might visit health care providers during the late summer months for a medical examination before the start of school, which represents a vaccination opportunity.
- **Pregnant women in the third trimester.** Vaccination during July and August can be considered for women who are in the third trimester of pregnancy during these months because vaccination has been associated in multiple studies with reduced risk for influenza illness in their infants during the first months after birth, when they are too young to receive influenza vaccine (*34*–*38*). For pregnant women in the first or second trimester during July and August, waiting until September or October to vaccinate is preferable, unless there is concern that later vaccination might not be possible.

### Selection of Vaccine

As in past years, various influenza vaccines will be available for the 2025–26 influenza season (Table 1). In addition to contraindications and precautions (Table 2) (Table 3), considerations for vaccine selection include the following recommendations from ACIP: 

**TABLE 1:** Influenza vaccines — United States, 2025–26 influenza season*

Vaccine type and trade name (manufacturer)	Presentation	Age indication	*μ*g HA (IIV3s and RIV3) or virus count (LAIV3) for each vaccine virus (per dose)	Route	Contains thimerosal as preservative
IIV3s (standard-dose, egg-based vaccines^†^)					
Afluria (Seqirus)	0.5-mL PFS^§^	≥3 yrs^§^	15 *μ*g/0.5 mL	IM^¶^	No
	5.0-mL MDV**	≥6 mos^§^ (needle and syringe) 18 through 64 yrs (jet injector)^¶^	7.5 *μ*g/0.25 mL 15 *μ*g/0.5 mL	IM^¶^	Yes 24.5 *μ*g Hg/0.5 mL**
Fluarix (GlaxoSmithKline)	0.5-mL PFS	≥6 mos	15 *μ*g/0.5 mL	IM^¶^	No
FluLaval (GlaxoSmithKline)	0.5-mL PFS	≥6 mos	15 *μ*g/0.5 mL	IM^¶^	No
Fluzone (Sanofi Pasteur)	0.5-mL PFS^††^	≥6 mos^††^	15 *μ*g/0.5 mL	IM^¶^	No
	5.0-mL MDV**	≥6 mos^††^	7.5 *μ*g/0.25 mL 15 *μ*g/0.5 mL	IM^¶^	Yes 25 *μ*g Hg/0.5 mL**
**ccIIV3 (standard-dose, cell culture–based vaccine)**					
Flucelvax (Seqirus)	0.5-mL PFS	≥6 mos	15 *μ*g/0.5 mL	IM^¶^	No
	5.0-mL MDV**	≥6 mos**	15 *μ*g/0.5 mL	IM^¶^	Yes 25 *μ*g Hg/0.5 mL**
**HD-IIV3 (high-dose, egg-based vaccine^†^)**					
Fluzone High-Dose (Sanofi Pasteur)	0.5-mL PFS	≥65 yrs	60 *μ*g/0.5 mL	IM^¶^	No
**aIIV3 (standard-dose, egg-based vaccine^†^ with MF59 adjuvant)**					
Fluad (Seqirus)	0.5-mL PFS	≥65 yrs	15 *μ*g/0.5 mL	IM^¶^	No
**RIV3 (recombinant HA vaccine)**					
Flublok (Sanofi Pasteur)	0.5-mL PFS	≥9 yrs	45 *μ*g/0.5 mL	IM^¶^	No
**LAIV3 (egg-based vaccine^†^)**					
FluMist (AstraZeneca)	0.2-mL prefilled single-use intranasal sprayer	2 through 49 yrs	10^6.5–7.5^ FFU/0.2 mL	Intranasal	No

**Abbreviations:** ACIP = Advisory Committee on Immunization Practices; aIIV3 = adjuvanted inactivated influenza vaccine, trivalent; ccIIV3 = cell culture–based inactivated influenza vaccine, trivalent; FDA = Food and Drug Administration; FFU = fluorescent focus units; HA = hemagglutinin; HD-IIV3 = high-dose inactivated influenza vaccine, trivalent; Hg = mercury; IIV3 = inactivated influenza vaccine, trivalent; IM = intramuscular; LAIV3 = live attenuated influenza vaccine, trivalent; MDV = multidose vial; PFS = prefilled syringe; RIV3 = recombinant influenza vaccine, trivalent.

* Manufacturer package inserts and updated CDC and ACIP guidance should be consulted for additional information including, but not limited to, indications, contraindications, warnings, precautions. Package inserts for U.S.-licensed vaccines are available from FDA at Vaccines Licensed for Use in the United States. Availability and characteristics of specific products and presentations might change or differ from what is described in this table and in the text of this report.

^†^ Although a history of severe allergic reaction (e.g., anaphylaxis) to egg is a labeled contraindication to the use of egg-based IIV3s and LAIV3, ACIP recommends that all persons aged ≥6 months with egg allergy should receive influenza vaccine and that any influenza vaccine (egg based or non–egg based) that is otherwise appropriate for the recipient’s age and health status can be used (see Persons with a History of Egg Allergy in Prevention and Control of Seasonal Influenza with Vaccines).

^§^ The approved dose volume for Afluria is 0.25 mL for children aged 6 through 35 months and 0.5 mL for persons aged ≥3 years. However, 0.25-mL PFSs are no longer available, and ACIP recommends that children aged ≤18 years, pregnant women, and all adults receive seasonal influenza vaccines only in single-dose formulations that are free of thimerosal as a preservative. The Afluria 0.5-mL PFS presentation should be used only for persons aged ≥3 years.

^¶^ IM-administered influenza vaccines should be administered by needle and syringe. Although the MDV presentation of Afluria is approved by FDA for administration via the PharmaJet Stratis jet injector for adults aged 18 through 64 years, ACIP recommends that children aged ≤18 years, pregnant women, and all adults receive seasonal influenza vaccines only in single-dose formulations that are free of thimerosal as a preservative. For older children and adults, the recommended site for IM influenza vaccination is the deltoid muscle. The preferred site for infants and young children is the anterolateral aspect of the thigh. Additional specific guidance regarding site selection and needle length for IM administration is available in the CDC General Best Practices for Immunization.

** An MDV formulation containing thimerosal might be available for the 2025–26 season. However, ACIP recommends that children aged ≤18 years, pregnant women, and all adults receive seasonal influenza vaccines only in single-dose formulations that are free of thimerosal as a preservative.

^††^ Fluzone is approved for children aged 6 through 35 months at either 0.25 mL or 0.5 mL per dose. However, 0.25-mL PFSs are no longer available, and ACIP recommends that children aged ≤18 years, pregnant women, and all adults receive seasonal influenza vaccines only in single-dose formulations that are free of thimerosal as a preservative. The Fluzone 0.5-mL PFS may be used for persons aged ≥6 months.

**TABLE 2:** Contraindications and precautions for the use of influenza vaccines — United States, 2025–26 influenza season*

Vaccine type	Contraindications	Precautions
Egg-based IIV3s	History of severe allergic reaction (e.g., anaphylaxis) to any component of the vaccine^†^ or to a previous dose of any influenza vaccine (i.e., any egg-based IIV, ccIIV, RIV, or LAIV)^§^	Moderate or severe acute illness with or without fever History of Guillain-Barré syndrome within 6 wks of receipt of influenza vaccine
ccIIV3	History of severe allergic reaction (e.g., anaphylaxis) to a previous dose of any ccIIV or any component of ccIIV3^§^	Moderate or severe acute illness with or without fever History of Guillain-Barré syndrome within 6 wks of receipt of influenza vaccine History of severe allergic reaction to a previous dose of any other influenza vaccine (i.e., any egg-based IIV, RIV, or LAIV)^¶^
RIV3	History of severe allergic reaction (e.g., anaphylaxis) to a previous dose of any RIV or any component of RIV3^§^	Moderate or severe acute illness with or without fever History of Guillain-Barré syndrome within 6 wks of receipt of influenza vaccine History of severe allergic reaction to a previous dose of any other influenza vaccine (i.e., any egg-based IIV, ccIIV, or LAIV)^¶^
LAIV3	History of severe allergic reaction (e.g., anaphylaxis) to any component of the vaccine^†^ or to a previous dose of any influenza vaccine (i.e., any egg-based IIV, ccIIV, RIV, or LAIV)^§^ Concomitant aspirin- or salicylate-containing therapy in children and adolescents^§^ Children aged 2 through 4 yrs who have received a diagnosis of asthma or whose parents or caregivers report that a health care provider has told them during the preceding 12 mos that their child had wheezing or asthma or whose medical record indicates a wheezing episode has occurred during the preceding 12 mos Children and adults who are immunocompromised due to any cause, including but not limited to immunosuppression caused by medications, congenital or acquired immunodeficiency states, HIV infection, anatomic asplenia, or functional asplenia (e.g., due to sickle cell anemia) Close contacts and caregivers of severely immunosuppressed persons who require a protected environment Pregnancy Persons with active communication between the CSF and the oropharynx, nasopharynx, nose, or ear, or any other cranial CSF leak Persons with cochlear implants** Receipt of the influenza antiviral medications oseltamivir or zanamivir within the previous 48 hours; receipt of peramivir within the previous 5 days; or receipt of baloxavir within the previous 17 days^††^	Moderate or severe acute illness with or without fever History of Guillain-Barré syndrome within 6 wks of receipt of influenza vaccine Asthma in persons aged ≥5 yrs Other underlying medical conditions that might predispose to complications after wild-type influenza infection (e.g., chronic pulmonary, cardiovascular [except isolated hypertension], renal, hepatic, neurologic, hematologic, or metabolic disorders [including diabetes mellitus])

**Abbreviations:** ACIP = Advisory Committee on Immunization Practices; ccIIV = cell culture–based inactivated influenza vaccine (any valency); ccIIV3 = cell culture–based inactivated influenza vaccine, trivalent; CSF = cerebrospinal fluid; IIV = inactivated influenza vaccine (any valency); IIV3 = inactivated influenza vaccine, trivalent; LAIV = live attenuated influenza vaccine (any valency); LAIV3 = live attenuated influenza vaccine, trivalent; RIV = recombinant influenza vaccine (any valency); RIV3 = recombinant influenza vaccine, trivalent.

* Manufacturer package inserts and updated CDC and ACIP guidance should be consulted for additional information including, but not limited to, indications, contraindications, warnings, and precautions. When a contraindication is present, a vaccine should not be administered. When a precaution is present, vaccination should generally be deferred but might be indicated if the benefit of protection from the vaccine outweighs the risk for an adverse reaction (General Best Practices for Immunization | CDC). Package inserts for U.S.-licensed vaccines are available from FDA at Vaccines Licensed for Use in the United States.

^†^ Although a history of severe allergic reaction (e.g., anaphylaxis) to egg is a labeled contraindication to the use of egg-based IIV3s and LAIV3, ACIP recommends that all persons aged ≥6 months with egg allergy should receive influenza vaccine and that any influenza vaccine (egg based or non–egg based) that is otherwise appropriate for the recipient’s age and health status can be used (see Persons with a History of Egg Allergy in Prevention and Control of Seasonal Influenza with Vaccines).

^§^ Labeled contraindication noted in package insert.

^¶^ If administered, vaccination should occur in a medical setting and should be supervised by a health care provider who can recognize and manage severe allergic reactions. Providers can consider consulting with an allergist in such cases to assist in identification of the component responsible for the allergic reaction.

** Injectable vaccines are recommended for persons with cochlear implant because of the potential for CSF leak, which might exist for a period after implantation. Providers might consider consultation with a specialist concerning risk for persistent CSF leak if an inactivated or recombinant vaccine cannot be used.

^††^ Use of LAIV3 in the context of influenza antivirals has not been studied; however, interference with activity of LAIV3 is biologically plausible, a possibility that is noted in the package insert for LAIV3. In the absence of data supporting an adequate minimum interval between influenza antiviral use and LAIV3 administration, the intervals provided are based on the half-life of each antiviral. The interval between influenza antiviral receipt and LAIV3 for which interference might occur might be further prolonged in the presence of medical conditions that delay medication clearance (e.g., renal insufficiency). Influenza antivirals might also interfere with LAIV3 if initiated within 2 weeks after vaccination. Persons who receive antivirals during the period starting with the specified time before receipt of LAIV3 through 2 weeks after receipt of LAIV3 should be revaccinated with an age-appropriate IIV3 or RIV3.

**TABLE 3:** Influenza vaccine contraindications and precautions for persons with a history of severe allergic reaction to a previous dose of an influenza vaccine* — United States, 2025–26 influenza season

Vaccine (of any valency) associated with previous severe allergic reaction (e.g., anaphylaxis)	Available 2025–26 influenza vaccine		
	Egg-based IIV3s and LAIV3	ccIIV3	RIV3
Any egg-based IIV or LAIV	Contraindication^†^	Precaution^§^	Precaution^§^
Any ccIIV	Contraindication^†^	Contraindication^†^	Precaution^§^
Any RIV	Contraindication^†^	Precaution^§^	Contraindication^†^
Unknown influenza vaccine	Allergist consultation recommended	Allergist consultation recommended	Allergist consultation recommended

**Abbreviations:** ACIP = Advisory Committee on Immunization Practices; ccIIV = cell culture–based inactivated influenza vaccine (any valency); ccIIV3 = cell culture–based inactivated influenza vaccine, trivalent; IIV = inactivated influenza vaccine (any valency); IIV3 = inactivated influenza vaccine, trivalent; LAIV = live attenuated influenza vaccine (any valency); LAIV3 = live attenuated influenza vaccine, trivalent; RIV = recombinant influenza vaccine (any valency); RIV3 = recombinant influenza vaccine, trivalent.

* Manufacturer package inserts and updated CDC and ACIP guidance should be consulted for additional information including, but not limited to, indications, contraindications, warnings, and precautions. Package inserts for U.S.-licensed vaccines are available from FDA at Vaccines Licensed for Use in the United States.

^†^ When a contraindication is present, a vaccine should not be administered, consistent with CDC’s General Best Practices for Immunization. In addition to the contraindications based on history of severe allergic reaction to influenza vaccines that are noted in the table, each individual influenza vaccine is contraindicated for persons who have had a severe allergic reaction (e.g., anaphylaxis) to any component of that vaccine. Vaccine components can be found in package inserts. Although a history of severe allergic reaction (e.g., anaphylaxis) to egg is a labeled contraindication to the use of egg-based IIV3s and LAIV3, ACIP recommends that all persons aged ≥6 months with egg allergy should receive influenza vaccine and that any influenza vaccine (egg based or non–egg based) that is otherwise appropriate for the recipient’s age and health status can be used (see Persons with a History of Egg Allergy in Prevention and Control of Seasonal Influenza with Vaccines).

^§^ When a precaution is present, vaccination should generally be deferred but might be indicated if the benefit of protection from the vaccine outweighs the risk for an adverse reaction, consistent with CDC’s General Best Practices for Immunization. Providers can consider using the following vaccines in these instances; however, vaccination should occur in an inpatient or outpatient medical setting with supervision by a health care provider who is able to recognize and manage severe allergic reactions: 1) for persons with a history of severe allergic reaction (e.g., anaphylaxis) to any egg-based IIV or LAIV of any valency, the provider can consider administering ccIIV3 or RIV3; 2) for persons with a history of severe allergic reaction (e.g., anaphylaxis) to any ccIIV of any valency, the provider can consider administering RIV3; and 3) for persons with a history of severe allergic reaction (e.g., anaphylaxis) to any RIV of any valency, the provider can consider administering ccIIV3. Providers can also consider consulting with an allergist to help determine which vaccine component is responsible for the allergic reaction.

- Children aged ≤18 years, pregnant women, and all adults should receive seasonal influenza vaccines only in single-dose formulations that are free of thimerosal as a preservative.
- All persons should receive an age-appropriate influenza vaccine (i.e., one that is approved for their age), with the exception that solid organ transplant recipients aged 18 through 64 years who are receiving immunosuppressive medication regimens may receive either trivalent high-dose inactivated influenza vaccine (HD-IIV3) or trivalent adjuvanted inactivated influenza vaccine (aIIV3) as acceptable options (without a preference over other age-appropriate IIV3s or trivalent recombinant influenza vaccine [RIV3]) (*8*,*9*).
- Except for vaccination for adults aged ≥65 years, ACIP makes no preferential recommendation for a specific vaccine when more than one licensed and recommended vaccine is available. Among adults aged ≥65 years any one of the following higher dose or adjuvanted influenza vaccines is preferentially recommended: HD-IIV3, RIV3, or aIIV3. If none of these three vaccines is available at an opportunity for vaccine administration, any other available age-appropriate influenza vaccine should be used (*4*,*5*).
- LAIV3 is not recommended during pregnancy, for immunocompromised persons, for persons with certain medical conditions, or for persons who are receiving, have recently received, or are about to receive influenza antiviral medications (Table 2). LAIV3 should not be administered to persons aged <2 or >49 years.

### Number of Doses, Route of Administration, and Dose Volumes

Children aged 6 months through 8 years who have received ≥2 previous doses of trivalent or quadrivalent influenza vaccine ≥4 weeks apart before July 1, 2025, should receive 1 dose of 2025–26 influenza vaccine. The previous 2 doses are not required to have been received during the same or consecutive influenza seasons. Children aged 6 months through 8 years who have not received ≥2 doses of trivalent or quadrivalent influenza vaccine administered ≥4 weeks apart before July 1, 2025, or whose influenza vaccination history is unknown, should receive 2 doses of 2025–26 influenza vaccine ≥4 weeks apart (Supplementary Figure). For children aged 8 years who require 2 doses of vaccine, both doses should be administered even if the child reaches age 9 years between receipt of dose 1 and receipt of dose 2. All persons aged ≥9 years need only 1 dose of a 2025–26 influenza vaccine.

Injectable influenza vaccines (i.e., inactivated and recombinant influenza vaccines) are administered intramuscularly. FDA-approved dose volumes are 0.5 mL for all age groups, with two exceptions (Table 1). The approved dose volume for Afluria (IIV3) is 0.25 mL per dose for children aged 6 through 35 months. The dose volume for children aged ≥3 years and adults is 0.5 mL per dose (*39*). The Afluria 0.5-mL prefilled syringes should not be administered to children aged <3 years, and 0.25-mL prefilled syringes are not available. Because use of multidose seasonal influenza vaccine formulations containing thimerosal as a preservative is no longer recommended, there is currently no ACIP-recommended formulation of Afluria for children aged 6 through 35 months.

The approved dose volume for Fluzone (IIV3) is either 0.25 mL or 0.5 mL per dose for children aged 6 through 35 months. Children aged ≥3 years and adults should receive 0.5 mL per dose (*40*). Although 0.25-mL prefilled syringes are not available, the Fluzone 0.5-mL prefilled syringes can be used for all persons aged ≥6 months. Use of multidose seasonal influenza vaccine formulations containing thimerosal as a preservative is no longer recommended.

### Contraindications and Precautions

Each influenza vaccine has a labeled contraindication for persons with a history of a severe allergic reaction (e.g., anaphylaxis) to any component of that vaccine (Table 2) (Table 3) (*11*,*12*,*39*–*45*). Vaccine components are listed in product package inserts. A history of severe allergic reaction (e.g., anaphylaxis) to egg is a labeled contraindication to the use of egg-based IIV3s and LAIV3, which might contain residual egg protein (*11*,*39*–*41*,*43*–*45*). However, ACIP recommends that all persons aged ≥6 months with egg allergy should receive influenza vaccine and that any influenza vaccine (egg based or non–egg based) that is otherwise appropriate for the recipient’s age and health status may be used (*6*,*7*). This recommendation was based on a review of evidence from 20 studies (16 of IIVs, one of virosomal influenza vaccine, and three of LAIV) that examined reactions after administration of seasonal influenza vaccines to egg-allergic persons via either full single-dose or split-dose administration protocols (13 of which reported inclusion of persons with a history of severe reaction or anaphylaxis to egg). No instances of anaphylaxis were reported (GRADE certainty level: very low) (*6*). Egg allergy alone necessitates no additional safety measures for influenza vaccination beyond those recommended for any recipient of any vaccine, regardless of severity of previous reaction to egg.

For persons who have had a severe allergic reaction (e.g., anaphylaxis) to a specific influenza vaccine, further receipt of that vaccine is contraindicated. Per package inserts, all egg-based IIV3s and LAIV3 are contraindicated for persons who have had a severe allergic reaction (e.g., anaphylaxis) to any influenza vaccine. Recommendations concerning consideration of non–egg-based influenza vaccines for such persons depends on the type of influenza vaccine associated with the previous severe allergic reaction (Table 2) (Table 3). Providers also might consider consulting with an allergist to help identify the vaccine component responsible for the reaction. Clinical settings in which vaccines are administered should be equipped to recognize and manage acute allergic reactions (*46*).

Moderate or severe acute illness with or without fever is a general precaution for vaccination (*46*). A history of Guillain-Barré syndrome within 6 weeks after receipt of a previous dose of influenza vaccine is considered a precaution for the use of all influenza vaccines (Table 2).

In addition to labeled contraindications for LAIV3 that are listed in the package insert, ACIP also considers several other conditions to be contraindications or precautions to the use of LAIV3 (Table 2). In addition, LAIV3 is not approved for and should not be given to persons aged <2 or >49 years.

## Recent Influenza Vaccine Labeling Changes

**FluMist (LAIV3).** In September 2024, FDA approved the nasal spray live attenuated influenza vaccine FluMist (LAIV3) for self-administration (for recipients aged 18 through 49 years) or administration by a caregiver aged ≥18 years (for children and adolescents aged 2 through 17 years) (*11*). FluMist for self-administration or caregiver administration is anticipated to become available during the 2025–26 season via the FluMist Home program, through which consumers provide information to determine their eligibility to order the vaccine (*47*). For persons who meet eligibility criteria to receive FluMist, vaccine will be shipped under temperature-controlled conditions to the address provided by the person placing the order. ACIP recommendations, contraindications, and precautions for use of FluMist for self-administration or caregiver administration are the same as those for health care provider administration (Table 2) (Table 3). FluMist will continue to be available for ordering and administration by health care providers.

**Flublok (RIV3).** In March 2025, FDA expanded approval of Flublok (RIV3), previously approved only for persons aged ≥18 years, to children and adolescents aged 9 through 17 years. The new labeled age indication for Flublok is ≥9 years (*12*). Approval was based on a nonrandomized open-label study comparing immunogenicity and safety of Flublok Quadrivalent (RIV4) among children and adolescents aged 9 through 17 years with immunogenicity and safety in adults aged 18 through 49 years (*12**,**48*). Flublok Quadrivalent met prespecified criteria for noninferiority of immunogenicity (lower limit of the two-sided 95% CI of the geometric mean titer ratios between age groups of >0.667 and lower limit of the two-sided 95% CI of the difference in seroconversion rates of >–10% at day 29 postvaccination for all four viral components). Among children and adolescents aged 9 through 17 years, the most common adverse reactions (occurring in ≥10% of participants) were injection site pain (34.4%), myalgia (19.3%), headache (18.5%), and malaise (16.1%) (*12*). Flublok is not approved or recommended for children aged <9 years.
